# Standard linkage and association methods identify the mechanism of four susceptibility genes for a simulated complex disease

**DOI:** 10.1186/1471-2156-6-S1-S142

**Published:** 2005-12-30

**Authors:** Nathan Pankratz, Ellen Edenberg, Tatiana Foroud

**Affiliations:** 1 Department of Medical and Molecular Genetics, Indiana University, School of Medicine, Indianapolis, IN, USA

## Abstract

The simulated dataset of the Genetic Analysis Workshop 14 provided affection status and the presence or absence of 12 traits. It was determined that all affected individuals must have traits E, F and H (EFH phenotype) and they must also have either trait B (B subtype) or traits C, D, and G (CDG subtype). A genome screen was performed, and linkage peaks were identified on chromosomes 1, 3, 5, and 9 using microsatellite markers. Dense panels of single-nucleotide polymorphism (SNP) markers were ordered for each of the four linkage peaks. In each case, association analyses identified a single SNP that accounted for the linkage evidence. The SNP on chromosome 1 appeared to primarily influence the B subtype, while the SNPs on chromosomes 5 and 9 primarily influenced the CDG subtype. The chromosome 3 SNP had the strongest effect and influenced both subtypes, as well as the requisite EFH phenotype. Recognizing the two subtypes prior to linkage analysis was key to identifying these loci using only a single replicate. This highlights the need in real life situations for careful examination of the phenotypic data prior to genetic analysis.

## Background

Studies designed to identify genes contributing to complex diseases have been ongoing for many years, utilizing different study designs and methods with varied success. The simulated dataset of the Genetic Analysis Workshop 14 (GAW14) provided an opportunity to evaluate the standard analysis techniques used to identify genes underlying complex phenotypes. We have also attempted to predict disease status given the presence of apparent "high risk" or deleterious alleles (those alleles associated with disease) identified in our analyses.

## Methods

All analyses were performed using the simulated dataset without knowledge of the underlying model, including number of genes or their location. Analyses were performed with complete genotypic and phenotypic data from replicate 1. The true model was obtained only at GAW14, and was used in this report to verify our findings. No additional results or figures were generated after learning the simulation model.

### Phenotype in populations

As the effect of genes may differ across subpopulations, each was analyzed separately as well as together. The linkage methods employed did not allow for large pedigrees; therefore, only the isolated populations – Aipotu (AI), Karangar (KA), and Danacaa (DA) – were used for linkage analysis. The large families recruited from the heterogeneous urban setting (NYC) were only used in secondary association analyses. The simulated dataset included a number of discrete traits as well as the affection status for the simulated disease. Presence or absence of specific traits was tabulated for each population among affecteds and unaffecteds. Disease subtypes based on these traits were assigned to each individual for future analyses.

### Genetic analysis

A genome screen was performed for affection status, as well as for each of the disease subtypes, using the nonparametric linkage analysis methods implemented in ALLEGRO [1]. To assist in prioritizing regions for further study, panels of dense single-nucleotide polymorphisms (SNPs) were purchased for those chromosomal locations with LOD scores greater than 2.0.

Association analyses were performed for each of the SNPs contained in these panels, using the transmission disequilibrium test and discordant sib-pair test implemented in the pedigree disequilibrium test (PDT) [2]. Tests were conducted for each of the subtypes identified above.

### Evaluation of penetrance

A single individual was randomly chosen from each family, and for each SNP the percentage of individuals with the corresponding phenotype was tabulated for those carrying 0, 1, and 2 deleterious alleles. This process was repeated 10,000 times and the bootstrapped mean penetrance is reported.

### Prediction of phenotype

Logistic regression was used to quantify the effects of the apparent deleterious alleles identified in these genetic analyses. Affection status and the subtypes were each used in turn as the dependent variable. The number of copies for each of the deleterious alleles was used as an independent variable. Interactions between genes and with sex were also added. Those variables without a significant contribution were dropped from the final predictive model.

## Results

### Phenotype in populations

Upon close inspection of affected individuals, it was discovered that all affected individuals had traits E, F, and H (the EFH phenotype). It was discovered that two subtypes existed. In addition to the EFH phenotype, affected individuals had to have either trait B (the B subtype) or they had to have traits C, D, and G (the CDG subtype). This rule held true for all populations; however, different populations had different ratios of the two subtypes. All affected individuals in the DA population had the B subtype, and all of the affected individuals in the KA population had the CDG subtype (see Table 1). Affected individuals in the AI and NYC populations had a mixture of the two subtypes. There was considerable overlap between the two subtypes in all populations.

### Genetic analyses

Linkage analyses identified four chromosomal regions with LOD scores greater than 2.0. Only one region, chromosome 3, was consistently seen in all subtypes and in all populations. Linkage to the region on chromosome 1 was only seen when analyzing the B subtype or the DA population. Conversely, the regions on chromosomes 5 and 9 were only seen in the CDG subtype and in the KA population (see Table 2).

Data for additional markers in these regions were obtained for follow-up studies. Packets 27, 28, and 29 were ordered for chromosome 1, packet 153 for chromosome 3, packet 207 from chromosome 5, and packet 417 for chromosome 9. Association studies were performed on all additional markers using the program PDT. A SNP on chromosome 3 (B03T3056) yielded the most significant result (*p*-value = 2.3 × 10^-20^) (see Table 3). Several SNPs near B03T3056 were also significantly associated with affection status, suggesting that this SNP was part of a "high-risk" haplotype. However, all of the information from the haplotypes was captured in B03T3056, suggesting that the ancestral haplotype had not fully decayed. Slight linkage disequilibrium (LD) was observed in this chromosomal region (D' = 0.68; r^2 ^= 0.16).

The deleterious allele could not be identified in the 3 packets directly under the chromosome 1 linkage peak when only affection status was examined. However, when the B subtype was used (recall that the strongest linkage to chromosome 1 was with the B subtype), B01T0561 was identified (*p*-value = 0.001). Similarly, the deleterious SNPs on chromosome 5 (B05T4136; *p*-value = 0.01) and chromosome 9 (B09T8333; *p*-value = 5.0 × 10^-7^) were identified using the CDG phenotype.

### Evaluation of penetrance

**Figure 1:**
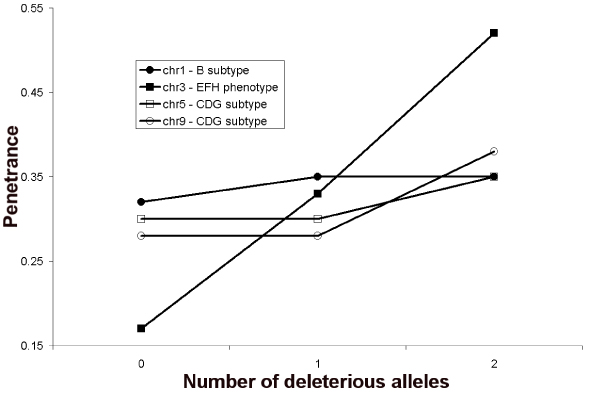
Estimated penetrance of identified SNPs for their corresponding phenotype.

Chromosome 3 (B03T3056) had the strongest contribution to disease risk, and its effect appeared additive (Figure 1). Chromosome 1 (B01T0561) had similar penetrances for those with 1 and 2 alleles and was later modeled as a dominant susceptibility gene. Chromosomes 5 (B05T4136) and 9 (B09T8333) had similar penetrances for those with 0 or 1 copy of the deleterious allele and were modeled as recessive susceptibility genes.

### Prediction of phenotype

Using these four identified SNPs, logistic regression was able to classify individuals as either affected or unaffected with 65.3% accuracy. When logistic regression was instead used to predict the subtypes and the predicted subtypes used to infer affection status, accuracy of disease classification remained the same. When the SNPs on chromosomes 1, 5, and 9 were modeled as additive (i.e., three levels: 0, 1, or 2 alleles) instead of dominant/recessive (i.e., two levels), several odds ratios decreased, but the predictive accuracy did not change. Interactions between genes or with sex were always non-significant. Similar levels of significance – 63.4% and 63.5% – were noted for replicates 2 and 3, when using the same beta values from the model obtained with replicate 1.

## Discussion

Analysis of the individual traits identified two subtypes of the simulated disease. All affected individuals had the EFH phenotype (i.e., all three discrete traits E, F, and H), but they could be classified into the B subtype (presence of trait B) or the CDG subtype (presence of all three traits C, D, and G). Three genome screens were performed, substituting the presence of these three phenotypes for affection status. Evidence of linkage differed depending on which subtypes were included as affected in the analysis. This observation matched closely to the true underlying disease model, unknown at the time, in which there were three ways an individual could become affected. The B subtype corresponds to the P1 phenotype. The CDG subtype corresponds to the P2 phenotype. And the third type detailed in the model (P3) required traits B, C, D, and G; however, since a subset of these traits were sufficient to cause disease, this was not recognized, and was not necessary to identify the underlying genes.

**Figure 2:**
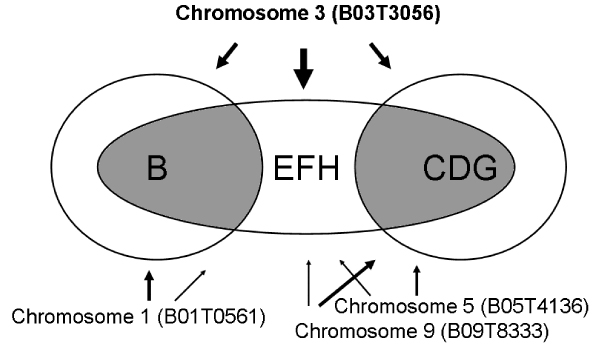
**Genotype/phenotype correlation**. Schematic of the relationship between the four identified SNPs and their corresponding phenotypes. A thicker arrow represents a stronger relationship. The shaded regions represent individuals that met the conditions to be considered affected.

We identified what we thought was the underlying disease polymorphism for each of the linkage regions on chromosomes 1, 3, 5, and 9. The SNP on chromosome 3 (B03T3056) had by far the strongest effect and exhibited an additive effect for all forms of the disease (i.e., having two copies was worse than one copy, which was worse than no copies of the deleterious allele). The SNP on chromosome 1 (B01T0561) appeared to act in an autosomal dominant manner, because there was no additional increase in risk if an individual had two copies of the deleterious allele versus just one. This SNP also appeared to affect only the B subtype and did not influence the CDG subtype. The SNPs on chromosomes 5 (B05T4136) and 9 (B09T8333) both appeared to follow an autosomal recessive mode of inheritance and both affected only the CDG subtype and not the B subtype. The disease model is summarized in the schematic of Figure 2.

The accuracy of classifying individuals as affected or unaffected based on these genotypes did not exceed 65.3%. This was originally interpreted as consistent with the existence of additional genes, environment and/or phenotypic variation not accounted for in the model. In fact, the major reason why the accuracy of disease classification was not greater was because the causative SNPs used in the simulation parameters to determine affection status were not provided in the fine mapping dataset. However, the "high-risk" haplotypes were identified, as well as the SNP that tagged these haplotypes most efficiently. Two additional modifier genes on different chromosomes were not identified (D5 and D6) due to their weak effect on the diagnostic phenotype.

Only five GAW14 studies correctly identified the major loci and haplotypes without having the "answers" or without grouping together all 100 replicates. Part of the reason for this is that a good understanding of the phenotype, namely identifying the two subtypes, was critical to reaching significance in any one replicate. Only two other groups correctly detailed these subtypes and all three discovered them in a different way. Our group tabulated the frequencies of the traits between affected and unaffected individuals, and performed genetic analyses after stratifying by subtype. Alternatively, MacGregor et al. incorporated qualitative covariates into their linkage analysis [3], while Liu et al. used machine learning to derive the patterns in the phenotype data [4].

## Conclusion

The ability to increase power to detect linkage based on minor phenotypic differences in a heterogeneous disease is a particularly important lesson to apply to real-world studies. A few groups were successful in identifying all four major disease loci because the ratio of subtypes was skewed in certain populations and because they analyzed these populations separately. However, this was only a proxy for the underlying subtypes and the same genes are not usually as influential among different isolated and admixed populations. The most important lesson from these results, obtained from simulated data, is that because phenotypic heterogeneity is often due to genotypic heterogeneity, as was the case here, it is vital that all available phenotypic information be analyzed thoroughly before genetic analyses are begun if the goal is to identify as many causal genes as possible.

## Abbreviations

AI: Aipotu

DA: Danacaa

GAW14: Genetic Analysis Workshop 14

KA: Karangar

LD: Linkage disequilibrium

PDT: Pedigree disequilibrium test

SNP: Single-nucleotide polymorphism

## Authors' contributions

NP designed the study, carried out the genetic analyses, and drafted the manuscript. EE helped with the genetic analyses. TF helped with the design of the study and helped to draft the manuscript. All authors read and approved the final manuscript.

## Figures and Tables

**Table 1:** Summary of subtypes within each population in replicate 1.

	% Affected			
	
Population	Total	B subtype	CDG subtype	EFH phenotype
Aipotu (AI)	41.1	65.1	62.6	100.0
Danacaa (DA)	36.9	100.0	10.9	100.0
Karangar (KA)	34.9	41.3	100.0	100.0
New York (NY)	29.1	71.5	59.9	100.0
				
All populations	34.9	69.9	57.8	100.0

**Table 2:** Genome screen results (LOD scores) for replicate 1

	Trait, all populations (position in cM)			Affection status (position in cM)			
		
Chr.	B subtype	CDG subtype	EFH phenotype	Aipotu (AI)	Danacaa (DA)	Karangar (KA)	All populations
1	**12.3 (162)**^**a**^	1.1	**5.6 (166)**	1.6	**5.4 (162)**	0.7	**5.5 (158)**
2	0.7	0.6	0.9	0.7	1.5	0.6	1
3	**3.5 (290)**	**2.6 (290)**	**4.4 (290)**	**3.7 (288)**	**2.2 (290)**	**3.5 (290)**	**9.3 (290)**
4	1	0.8	1.2	1.3	1	1.5	1.2
5	1.2	**4.8 (7)**	**3.7 (7)**	0.9	1.2	**4.8 (1)**	**3.6 (1)**
6	1.1	1.6	1	1	1	1.1	1.1
7	1.6	1	0.8	1.1	1.4	0.7	0.9
8	0.6	1.1	0.5	0.4	1	0.6	0.5
9	1.7	**4 (8)**	**2.5 (9)**	0.7	1.9	**4.5 (2)**	**2.5 (5)**
10	1	1.5	0.6	0.6	0.5	1.2	0.6

^a^All suggestive (> 2.2) and significant (≥ 3.6) LOD scores are bold.

**Table 3:** Association results for the 4 deleterious SNPs identified in replicate 1

				Average PDT chi-square value (*p*-value)			
				
Chr	Packet	SNP	disease allele	affection status	B subtype	CDG subtype	EFH phenotype
1	029	B01T0561	2	1.5 (0.22)	**10.5 (10**^**-3**^**)**	0.5 (0.47)	0.3 (0.61)
3	153	B03T3056	1	85.5 (2.3 × 10^-20^)	55.2 (10^-12^)	44.3 (3 × 10^-11^)	**85.6 (2 × 10**^**-20**^**)**
5	207	B05T4136	1	2.4 (0.12)	1.5 (0.22)	**6.4 (0.01)**	1.6 (0.21)
9	417	B09T8333	2	14.8 (10^-4^)	1.1 (0.30)	**25.3 (5 × 10**^**-7**^**)**	14.7 (10^-4^)

^a^The most significant *p*-value for each chromosome is bolded.
